# Supplementary material for: Peripheral Nerve Transection Predominantly Drives Sympathetic Nerve Sprouting in Mouse Dorsal Root Ganglia
Source: bioRxiv. 2025 Nov 10:2025.11.07.687300. Preprint. [Version 1] doi: 10.1101/2025.11.07.687300 (PMC12642574; doi:10.1101/2025.11.07.687300)
Supplement: Supplement 1 [file media-1.pdf]

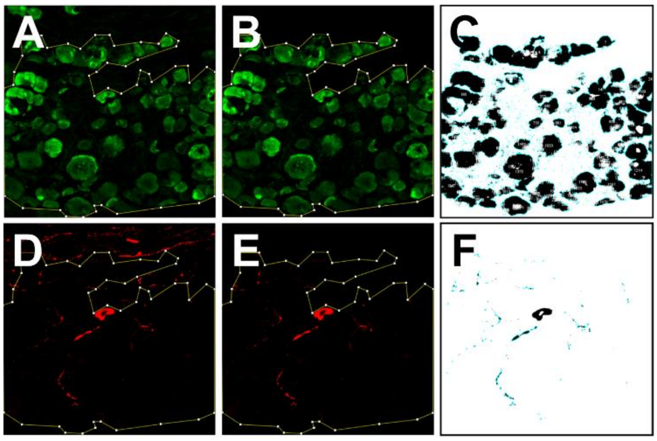

Supplemental figure 1. Analysis methods of the innervation density of TH-IR fiber.

(A) NeuN+ regions were manually delineated using ImageJ software. **(B)** Signals outside these regions were excluded using the “Clear Outside” plugin. **(C)** Particle analysis was performed for NeuN+ regions (green) after adjusting the threshold. **(D)** The identified NeuN+ region areas were applied to the TH+ channel (red). **(E)** Signals outside the NeuN+ regions were excluded using the “Clear Outside” plugin. **(F)** Particle analysis of TH-IR fibers was conducted after threshold adjustment, with a size range of 1-100 pixels^2 set to exclude signals from TH+ neurons. TH, Tyrosine hydroxylase; IR, Immunoreactive

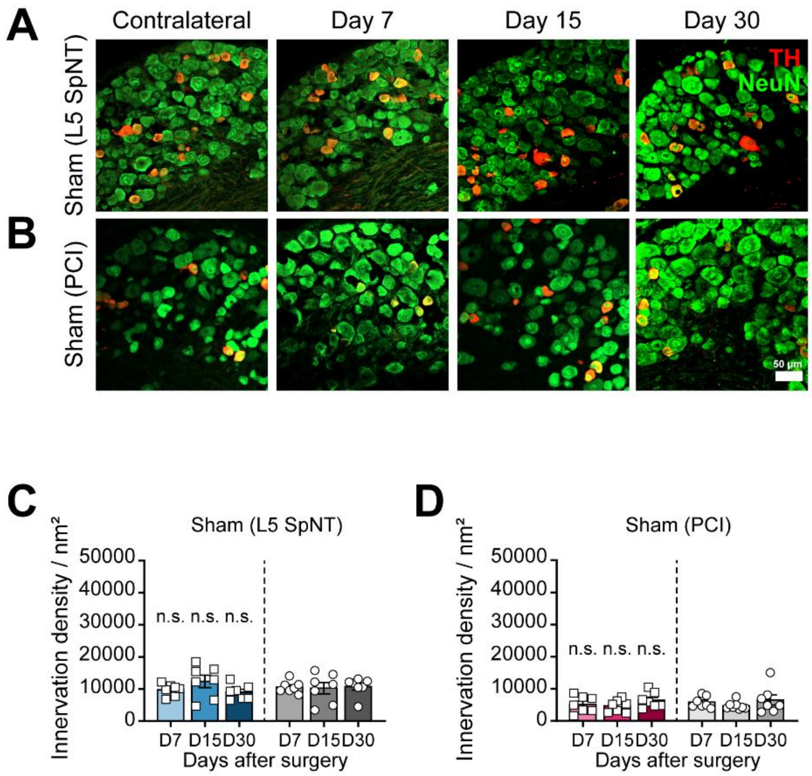

Supplemental figure 2. TH-IR fibers are not sprouted into DRGs following sham surgery of L5 SpNT and PCI.

(A-D) Representative immunostaining images and quantitative analysis of TH-IR fibers (nm^2) (red) in NeuN+ DRG neurons (μm^2) (green) at 7, 15, and 30 days after Sham surgery of L5 SpNT and PCI. Data represent mean ± SEM (n=7-8 mice per group, average of 5 sections per mouse); two-tailed unpaired *t*-test. TH, Tyrosine hydroxylase; IR, Immunoreactive; DRG, Dorsal root ganglia; L5 SpNT, L5 Spinal nerve transection; PCI, Partial sciatic nerve crush injury; n.s., not significant

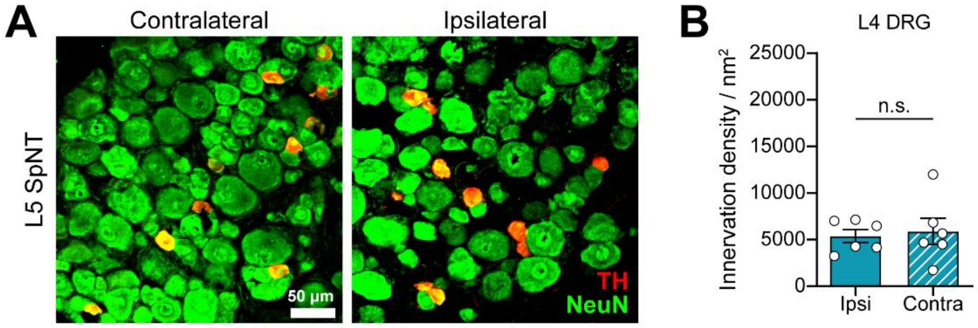

Supplemental figure 3. TH-IR fibers are not sprouted into L4 DRG following L5 SpNT.

(A, B) Representative immunostaining images and quantitative analysis of TH-IR fibers (nm^2) (red) in NeuN+ DRG neurons (μm^2) (green) in L4 DRG at 15 days after L5 SpNT. TH-IR fibers to ipsilateral L4 DRG were not observed after L5 SpNT. Data represent mean ± SEM (n=6 mice per group, average of 5 sections per mouse); Two-tailed unpaired *t*-test. TH, Tyrosine hydroxylase; IR, Immunoreactive; DRG, Dorsal root ganglia; L5 SpNT, L5 Spinal nerve transection; n.s., not significant

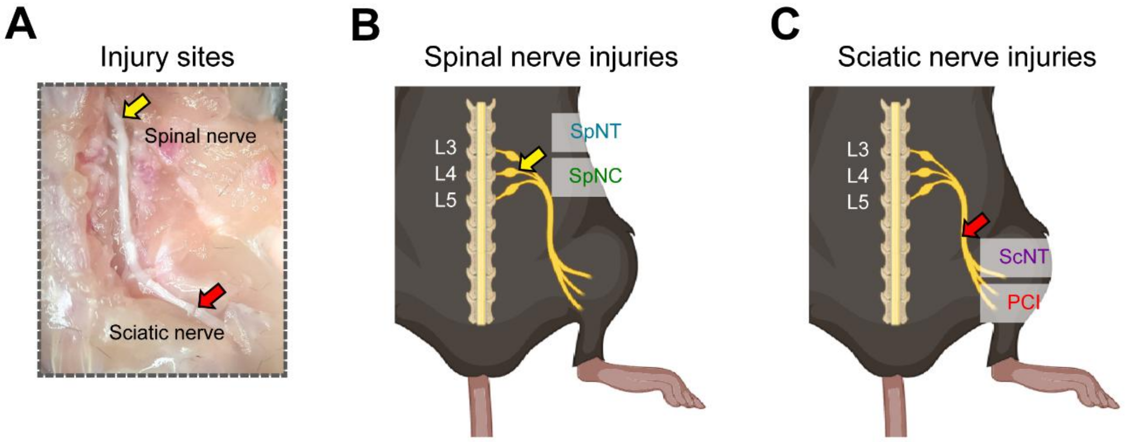

Supplemental figure 4. Schematic images of L4 SpNT, L4 SpNC, ScNT and PCI indicate the variations in the location and type of nerve injury.

(A) Injury sites of spinal nerve injuries (yellow arrow) and sciatic nerve injuries (red arrow) in WT mice. **(B)** Schematic image of L4 SpNT and L4 SpNC. **(C)** Schematic image of ScNT and PCI. WT, wild type; L4 SpNT, L4 Spinal nerve transection; L4 SpNC, L4 Spinal nerve crush; ScNT, Sciatic nerve transection; PCI, Partial sciatic nerve crush injury

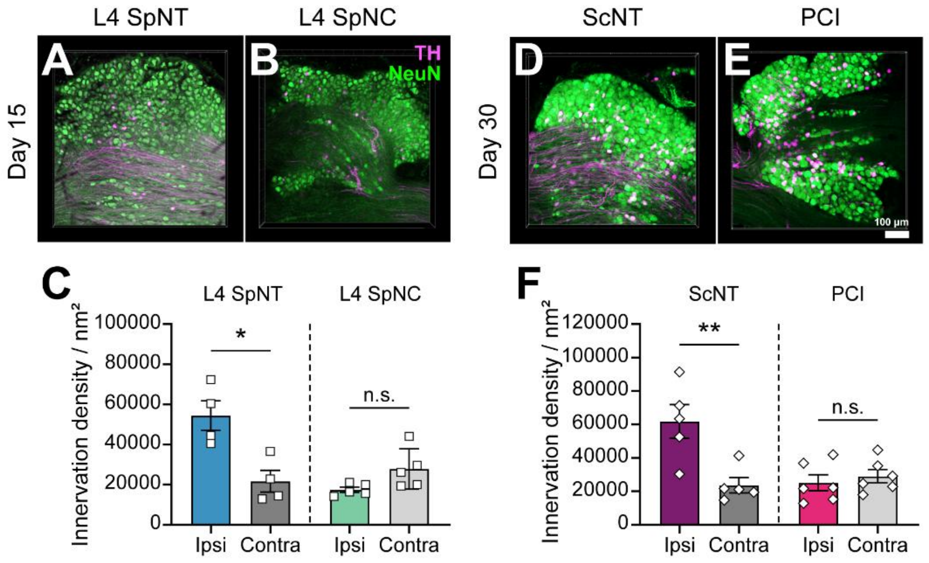

Supplemental figure 5. TH-IR fiber sprouting depends on injury type rather than injury location.

(A-C) Representative whole-mount immunostaining and quantification of TH-IR fibers (magenta) in NeuN+ DRG neurons (green) at 15 days following SpNC and L4 SpNT. Significant sympathetic fiber sprouting was observed only in the SpNT model (n=4–5 mice per group; average of 5 sections per mouse). **(D-F)** Whole-mount staining of L4 DRG following PCI and ScNT. Significant TH+ sympathetic sprouting occurred exclusively after transection injuries. (n=5–6 mice per group; average of 5 sections per mouse). Data represent mean ± SEM; *$p < 0.05$, **$p < 0.01$, two-tailed unpaired t-test. L4 SpNT, L4 Spinal nerve transection; L4 SpNC, L4 Spinal nerve crush; ScNT, Sciatic nerve transection; PCI, Partial sciatic nerve crush injury; n.s., not significant

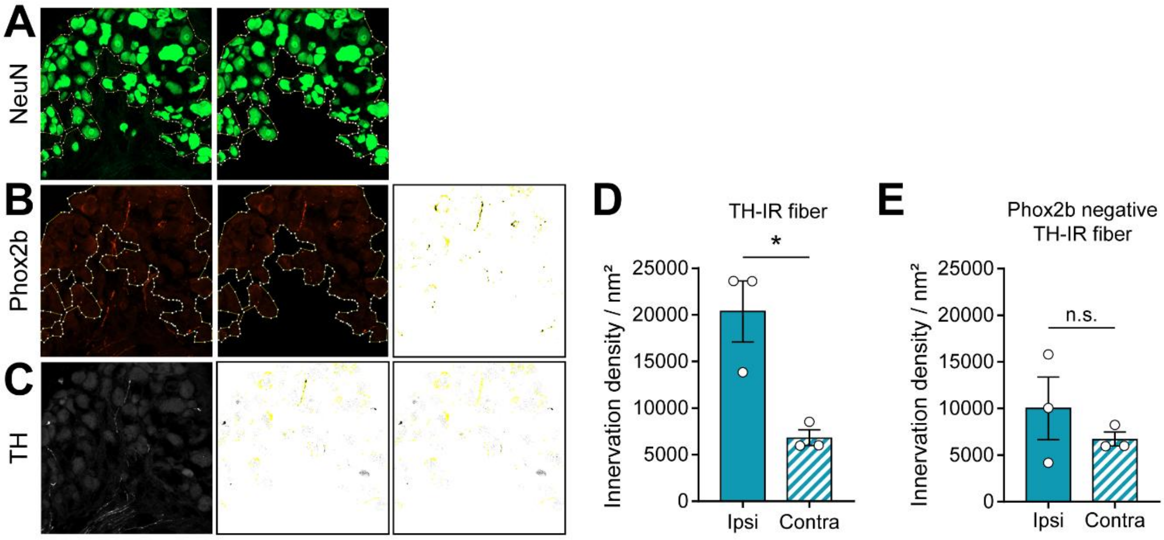

Supplemental figure 6. Phox2b-negative and TH-IR fibers are not increased after L4 SpNT.

(A) NeuN+ regions (green) were manually delineated using Image J software. **(B)** The area of Phox2b+ fibers (red) within the NeuN+ region was measured. **(C)** The ROI of Phox2b+ regions was excluded from TH-IR regions using the "Fill" plugin. **(D)** The innervation density of TH-IR fibers within the NeuN+ region was analyzed 15 days after L4 SpNT in Phox2b-tdTomato mice. Data represent mean ± SEM (n=3 mice per group, average of 5 sections per mouse); *$p < 0.05$, two-tailed unpaired *t*-test. **(E)** The innervation density of Phox2b- and TH+ fibers within the NeuN+ region was analyzed 15 days after L4 SpNT in Phox2b-tdTomato mice. Data represent mean ± SEM (n=3 mice per group, average of 5 sections per mouse); two-tailed unpaired *t*-test. ROI, Region of interest; TH, Tyrosine hydroxylase; IR, Immunoreactive; L4 SpNT, L4 Spinal nerve transection; n.s., not significant

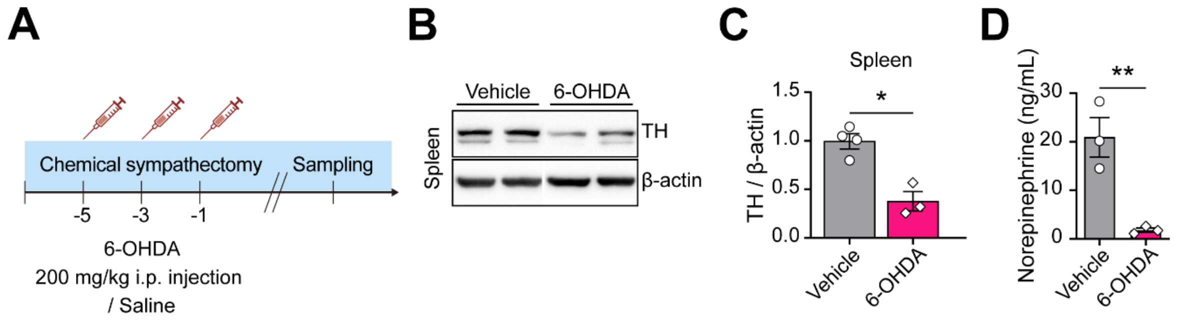

Supplemental figure 7. The level of TH and NE in the spleen decreased in mice treated with 6-OHDA.

(A) A schematic representation of injection and sampling schedule. **(B, C)** Validation of chemical sympathectomy a day after the injection of 6-OHDA (200mg/kg, *i.p.*). The protein level of TH significantly decreased in spleen of the 6-OHDA injected mice. Data represent mean ± SEM (n=3-4 mice per group); **p* < 0.05, two-tailed unpaired *t*-test. **(D)** Level of NE also significantly decreased in the spleen of 6-OHDA-treated mice. Data represent mean ± SEM (n=3 mice per group); ***p* < 0.01, two-tailed unpaired *t*-test. 6-OHDA, 6- Hydroxydopamine; *i.p.*, intraperitoneal; TH, Tyrosine hydroxylase; NE, Norepinephrine

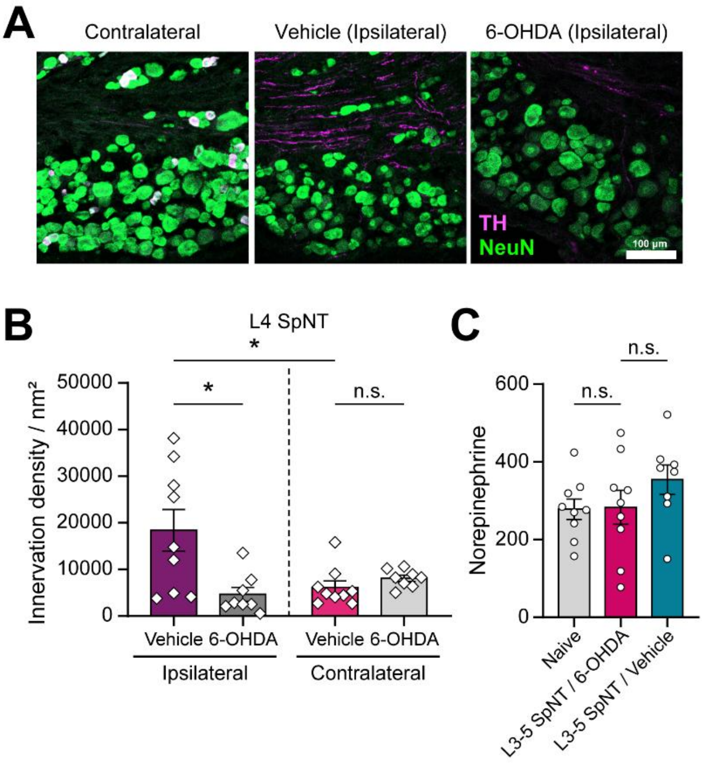

Supplemental figure 8. The level of TH in the DRG decreased in mice treated with 6-OHDA.

(**A, B**) Representative immunostaining images and quantitative analysis of TH-IR fibers (nm^2) (red) in NeuN+ DRG neurons (μm^2) (green) in 6-OHDA- vs. vehicle-treated mice at 15 days after L4 SpNT. Data represent mean ± SEM (n=8-9 mice per group, average of 5 sections per mouse); *$p < 0.05$, two-tailed unpaired *t*-test. (**C**) Quantification of NE levels in L3–L5 DRGs in 6-OHDA- *vs.* vehicle-treated mice at 15 days after L3-5 SpNT. NE levels tended to be lower in 6-OHDA–treated mice compared with vehicle-treated groups, although the difference was statistically not significant. Data represent mean ± SEM (n=8-9 mice per group; pooled samples of two mice per group); two-tailed unpaired *t*-test. 6-OHDA, 6-Hydroxydopamine; TH, Tyrosine hydroxylase; L4 SpNT, L4 Spinal nerve transection; NE, Norepinephrine; n.s., not significant

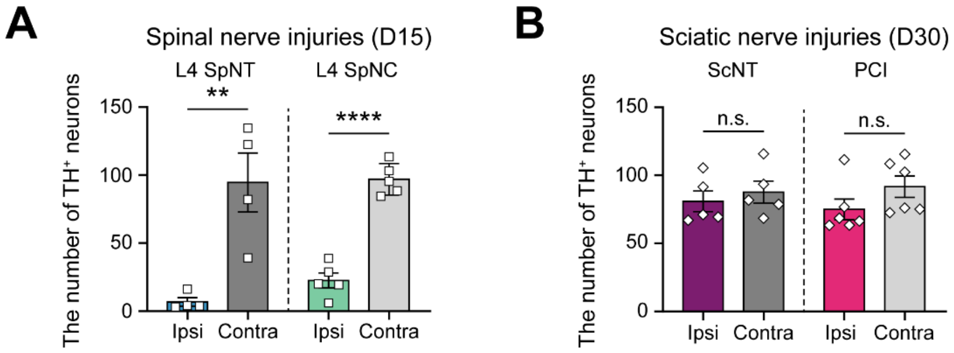

Supplemental figure 9. The number of TH+ DRG neurons following L4 SpNT, L4 SpNC, ScNT and PCI.

(A) The number of TH+ DRG neuron at 15 days after L4 SpNT and L4 SpNC. The number of TH+ neurons in ipsilateral DRG decreased following both injuries. Data represent mean ± SEM (n=4-5 mice per group, average of 5 sections per mouse); **p < 0.01, ****p < 0.0001, two-tailed unpaired t-test. **(B)** The number of TH+ DRG neuron at 30 days after ScNT and PCI. The number of TH+ neurons was not changed in ipsilateral DRG following both injuries. Data represent mean ± SEM (n=4-5 mice per group, average of 5 sections per mouse); two-tailed unpaired t-test. TH, Tyrosine hydroxylase; DRG, Dorsal root ganglia; L4 SpNT, L4 Spinal nerve transection; L4 SpNC, L4 Spinal nerve crush; ScNT, Sciatic nerve transection; PCI, Partial sciatic nerve crush injury; n.s., not significant

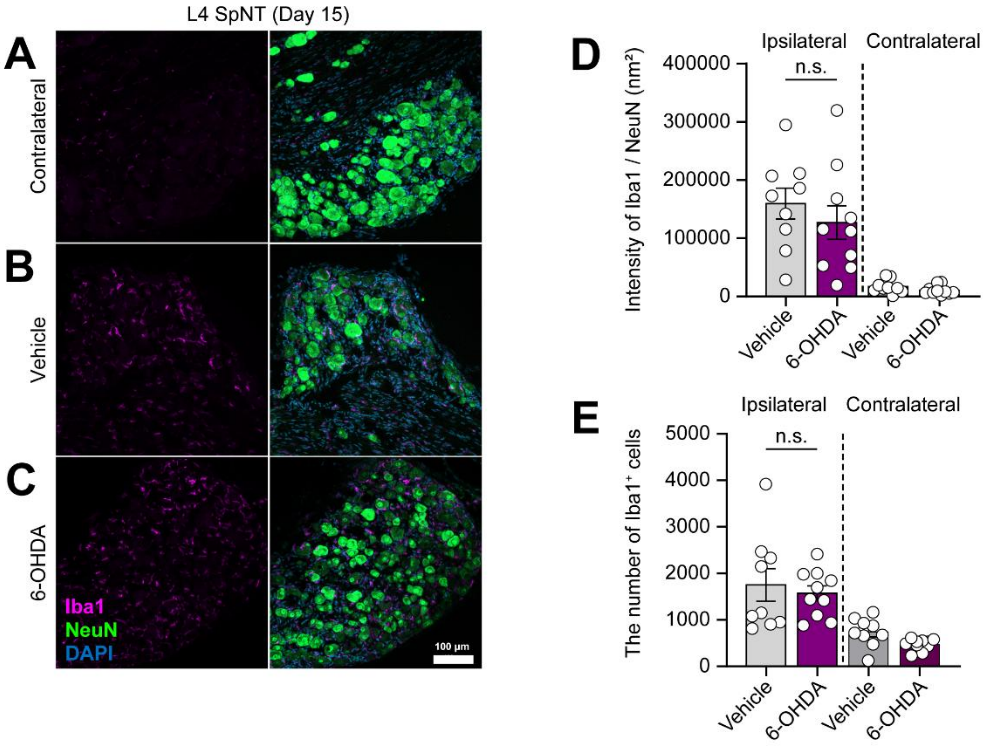

Supplemental figure 10. The intensity of Iba1+ macrophages is not significantly decreased following 6-OHDA treatment in L4 SpNT mice.

(A, B, C) Representative immunostaining images and quantitative analysis of Iba1-IR macrophages (nm^2) (magenta) in NeuN+ DRG neurons (μm^2) (green) in 6-OHDA- *vs.* vehicle-treated mice at 15 days after L4 SpNT. Data represent mean ± SEM (n=9-10 mice per group, average of 5 sections per mouse); two-tailed unpaired *t*-test. IR, Immunoreactive; 6-OHDA, 6- Hydroxydopamine; L4 SpNT, L4 Spinal nerve transection; n.s., not significant
